# Evaluating the impact of a ‘virtual clinic’ on patient experience, personal and provider costs of care in urinary incontinence: A randomised controlled trial

**DOI:** 10.1371/journal.pone.0189174

**Published:** 2018-01-18

**Authors:** Georgina Jones, Victoria Brennan, Richard Jacques, Hilary Wood, Simon Dixon, Stephen Radley

**Affiliations:** 1 Department of Psychology, School of Social Sciences, Leeds Beckett University, Leeds, United Kingdom; 2 Health Economics and Decision Science, School of Health & Related Research, University of Sheffield, Sheffield, United Kingdom; 3 Design, Trials and Statistics, School of Health & Related Research, University of Sheffield, Sheffield, United Kingdom; 4 Urogynaecology Unit, Jessop Wing, Sheffield Teaching Hospitals NHS Trust, Sheffield, United Kingdom; Medway NHS Foundation Trust, UNITED KINGDOM

## Abstract

**Objective:**

To evaluate the impact of using a ‘virtual clinic’ on patient experience and cost in the care of women with urinary incontinence.

**Materials and methods:**

Women, aged > 18 years referred to a urogynaecology unit were randomised to either (1) A Standard Clinic or (2) A Virtual Clinic. Both groups completed a validated, web-based interactive, patient-reported outome measure (ePAQ-Pelvic Floor), in advance of their appointment followed by either a telephone consultation (Virtual Clinic) or face-to-face consultation (Standard Care). The primary outcome was the mean ‘short-term outcome scale’ score on the Patient Experience Questionnaire (PEQ). Secondary Outcome Measures included the other domains of the PEQ (Communications, Emotions and Barriers), Client Satisfaction Questionnaire (CSQ), Short-Form 12 (SF-12), personal, societal and NHS costs.

**Results:**

195 women were randomised: 98 received the intervention and 97 received standard care. The primary outcome showed a non-significant difference between the two study arms. No significant differences were also observed on the CSQ and SF-12. However, the intervention group showed significantly higher PEQ domain scores for Communications, Emotions and Barriers (including following adjustment for age and parity). Whilst standard care was overall more cost-effective, this was minimal (£38.04). The virtual clinic also significantly reduced consultation time (10.94 minutes, compared with a mean duration of 25.9 minutes respectively) and consultation costs compared to usual care (£31.75 versus £72.17 respectively), thus presenting potential cost-savings in out-patient management.

**Conclusions:**

The virtual clinical had no impact on the short-term dimension of the PEQ and overall was not as cost-effective as standard care, due to greater clinic re-attendances in this group. In the virtual clinic group, consultation times were briefer, communication experience was enhanced and personal costs lower. For medical conditions of a sensitive or intimate nature, a virtual clinic has potential to support patients to communicate with health professionals about their condition.

## Introduction

One initiative to improve the efficiency and accessibility of outpatient care has been the implementation of ‘virtual clinics’. At the heart of a virtual clinic is the use of telemedicine to support the assessment, monitoring and management at a distance, away from traditional face to face clinic consultation [1].

Virtual clinics have been advocated for non-complex cases and have been implemented for the management of new [2] and follow-up patients [3–4] and for different specialties in both primary [5–6] and secondary care [7–8]. Potential patient benefits include improved satisfaction, reduced carbon footprint and reduction in unnecessary appointments and journeys to hospital [9]. The format and delivery of a virtual clinic varies widely depending upon the technology deployed including telephone [9], online web sessions [6] and Skype [5].

ePAQ-PF (electronic Personal Assessment Questionnaire-Pelvic Floor) is a validated, web-based interactive patient-reported outcome measure which offers in-depth evaluation of a woman’s pelvic floor symptoms and their impact on quality of life [10]. Previous research in primary and secondary care has established the psychometric properties of the instrument [11–13] and the system architecture for which enables patients to securely and anonymously complete a detailed, interactive web-based assessment.

Qualitative research involving home completion of ePAQ-PF, suggested positive patient experiences. These were particularly in terms of helping women to understand their condition, improve communication and prepare them for consultations, [14]. A randomised study in the USA observed increased discussion rates relating to intimate conditions in women randomised to use ePAQ [15]. It was therefore hypothesized that the assessment provided by ePAQ-PF could be used to support telephone consultations in a ‘virtual clinic’. However, such a development warranted scrutiny in terms of patient experience and cost [16].

We conducted a randomised controlled trial to evaluate the impact of a virtual clinic (ePAQ-PF completion) followed by telephone consultation). Our objectives were to compare patient experience and cost outcomes in new patients referred to the urogynaecology service who utilised the virtual clinic, with women who attended for standard care in the out-patient department.

## Materials and methods

This trial was designed as a randomised, parallel group trial. The study protocol was approved by the North Sheffield ethics committee and is registered at www.clinicaltrials.gov (identifier NCT02176330). The trial was retrospectively registered because the first participant was recruited into the study in June 2008 before the date when prospective registration was mandatory. Therefore, it did not become apparent that trial registration was necessary until analysing the data and preparing the manuscript for publication. The trial protocol (see S1 File Study Protocol) and supporting CONSORT checklist (see S2 File CONSORT Statement) are available as supporting information.

### Participants

Participant recruitment and follow-up took place between June 2008 and Oct 2010. All women referred to urogynaecology services at Sheffield Teaching Hospitals (STH), aged ≥ 18 years and able to read and understand English between July 2008 and April 2010 were eligible for study entry. Potential participants were identified by review of referral letters received in the clinic to which the patient had been referred. Referral letters were screened by senior medical staff involved in the study: Women in whom physical examination and urinalysis had not been carried out and documented by the referring clinician or GP, or those women in whom physical examination was considered essential at initial consultation (e.g. women with prolapse) were excluded from the study. When follow-up arrangements were made, such as for outpatient clinic, physiotherapy or urodynamics, it was clearly documented that pelvic examination may be required for virtual clinic patients.

Patients considered suitable were then contacted by telephone by the research nurse to discuss the study and those interested in participating were then sent a study information leaflet and consent form.

Women who did not reply within 1 week were contacted by telephone to assess whether they wished to participate in the study. Those women who could not be contacted at this point, or who declined study entry were sent a standard outpatient clinic appointment. To prevent unwarranted delays, patients who were not contactable by telephone within 48 hours of the reciept of the referral letter were excluded from the study and sent a standard outpatient clinic appointment, as were patients who expressed no interest in participating in the study.

#### Control group: Usual care

All women randomised to usual care were posted appointment details to attend the urogynaecology clinic. ‘Usual care’ included the option of completing the ePAQ on arrival in clinic, immediately prior to the clinical consultation. As is currently standard practice, the results of the ePAQ were used to inform and support clinical assessment, however, as the ePAQ-PF is completed *immediately* prior to the clinical consultation, patients were not triaged or provided with any additional information on the basis of their questionnaire results.

#### Intervention group: Virtual clinic

All women randomised to the virtual clinic were posted information details and a voucher letter inviting them to complete the questionnaire on-line. This letter includes details of the web address for ePAQ-online (www.epaq-voucher.co.uk) with information explaining how to access and use the website. On completion of the questionnaire, each patient had the option of printing her questionnaire report, which included a summary of symptom scores in each area (urinary, bowel, vaginal and sexual).

The same clinicians were involved in the outpatient department and therefore a virtual clinic and a standard approach was agreed: Confirming patient name, date of birth and expectation and willingness to undergo telephone consultation. No answer-machine messages were left if patients did not answer the telephone. All virtual clinic patients were offered the opportunity to attend in person, including for physical examination, if this was not already deemed necessary on conclusion of the telephone consultation. Patients in both standard care and telephone consultation were routinely offered a copy of correspondence, including any correspondence to their GP. The virtual clinic was based on telephone consultation, which is commonly used in patient care and no formal telemedicine training was received.

All data were encrypted and anonymised (in line with data protection guidelines) and were only personalised to the individual when digitally transferred to the password protected server on the N3 network. The clinician used the ePAQ-PF report in conjunction with the patient’s own casenotes and original referral letter to support the subsequent telephone consultation (this constituted the Virtual Clinic). The research nurse then arranged their telephone consultation. Women in this group who felt unable to complete the questionnaire online would use this telephone call with the research nurse to make arrangements to attend and complete the questionnaire at the hopsital.

### Outcome measures

In order to achieve uniformity of approach (between the Virtual Clinic and the control group) valdiated outcome questionnaires; Patient Experience Questionnaire (PEQ [17] and Client Satisfaction Questionnaire-8 (CSQ-8) [18] were posted to patients *no later than two weeks* following their first clinic consultation (baseline). Women were asked to complete and return these by post. All subjects were also asked to complete the QQ10 [19] (posted no later than two weeks after the first clinic consultation). These were analysed to evaluate any differences between groups. In addition, a subsample of patients were also asked to complete the Short Form-12 (SF-12) [20] at baseline and 6 months afterwards. The reason that only a subsample of patients were asked to complete this was because in error, the SF-12 was omitted from the questionniares to be completed at baseline. Once this was realised it was sent out. This meant there were only those patients included with complete SF-12 data (at baseline and 6 months) included in the analysis.

#### The PEQ

The PEQ was developed to measure patient experience of a general practice consultation [17]. It contains 18 items scored in four domains: 1) Communication, 2) Emotions 3) Short-term outcome, 4) Barriers and 5) Auxillary Staff. As this instrument was to be used to assess virtual and secondary care consultations, only the first 4 domains (contining 16 items) were considered relevant to this study. Domains 1, 3 and 4 are scored from 1 to 5, and the emotion scale is scored between 1 to 7. For each of these domains, each patient’s responses were summed, and then the mean for that domain calculated. Higher scores represent a positive patient experiences relating to communication, emotions, consultation outcome and communication barriers.

#### The CSQ-8

The CSQ-8 is a validated instrument which contains 8 items measuring the patient’s satisfaction following receiving a service. It is scored by simply summing the individual item scores which generates an overall score between 8 to 32. A higher score indicates more patient satisfaction [18].

#### QQ-10

The QQ10 (The Questionnaire’s Questionniare 10) is a 10-item instrument specifically designed for evaluating patients views on the use of a questionnaire as part of their healthcare. The 10 items are scored using a 5-point Likert rating from strongly agree to strongly disagree (coded as 0–4) which are then summed to produce two domains relating to the value and burden of using the instrument during a healthcare episode. These two domains are calculated by summing up scores of the first six questions and of the last four questions respectively based upon the patient’s repsonses. The higher the scores for the Value domain the more valuable and positive the new questionniare is perceived; and the higher the scores for the Burden domain, the more burdensome the new questionniare is perceived by participants [19].

#### ePAQ-PF

ePAQ-PF consists of 120 items and 19 scored domains covering the four key dimensions of pelvic floor health: Urinary, Bowel, Vaginal and Sexual. Each domain is summed and then converted on to a scale from 0–100 where 0 indicates perfect health and 100 the worst possible health status [11–13].

#### The SF-12

The SF-12 is a standardised, multidimensional, generic measure of health related quality of life (HRQOL) [20]. The results were converted into utility values using the SF-6D algorithm (Brazier & Roberts 2004). These values are anchored on a scale of 0 (death) to 1 (full health) which represent individuals preference for a health condition. These values are then combined with their timings to calculate an area under the curve, which represents quality adjusted life years (QALY) which were employed in the cost utility analyses [21]. The area under the curve was estimated using the trapezium rule [22].

#### Number and type of referrals

At 6-month follow-up the number and type of primary and secondary care referrals made on behalf of patients in both arms of the study was recorded using a standard proforma.

### Cost effectiveness

The use of ePAQ online prior to clinic appointments and the move away from the traditional consultation to a telephone consultation could potentially have significant economic impact, whilst also substantially changing patients’ experience of care. Therefore, an economic analysis was undertaken alongside the clinical trial to determine whether the use of ePAQ online in combination with telephone consultation was cost-effective when compared with standard care. The study estimated the costs of providing assessment and care to six months in both arms of the study, and cost-effectiveness by an incremental cost per QALY. Analyses were performed from two perspectives; (1) the cost to the NHS and (2) a broader societal cost that included patient expenditure and lost productivity. Due to the six month timeframe, no discounting of costs of effects was indicated. An economic evaluation was undertaken alongside the trial in line with good practice guidelines [23] and reported according to the Consolidated Health Economic Evaluations Reporting Standards (CHEERS) statement [24].

#### Resource use

Resource use data were collected at two time points: (1) Initial consultation and (2) Six month follow-up. A micro-costing study was undertaken on a sub-set of patients to derive costs for initial ePAQ completion and consultation; for these patients their consultation was timed and costs for staff time, computers, software and overheads were applied. Six months following study recruitment, patients were posted a questionnaire asking for resources used since their consultation. Resource use data included personal expenditure relating to (1) Bladder, bowel or vaginal problems; (2) Time off work; (3) Time away from usual activities; (4) Visits to the general practitioner, nurse or other health professionals, and (5) Inpatient and outpatient visits. The proforma also recorded details of prescribed medication, however, most patients included this within personal expenditure and due to the inconsistent reporting of prescription data, these costs were excluded from the economic analysis.

UK unit costs were applied to resource use estimates for patients in each study arm. All costs were inflated to 2011 United Kingdon (UK) prices using the pay and prices index [25] and value-added tax (VAT) was excluded in line with NICE guidance [26]. Staff time was costed using the *Unit Costs of Health and Social Care* publication and included overhead costs [25]. Costs of surgical procedures were estimated using a per-elective inpatient episode cost based on the National Schedule of Reference Costs [27].

### Statistical power

The primary outcome for the purpose of sample size determination was the mean outcome scale sore on the PEQ completed after first clinical consultation. Steine et al [17] reported a mean score of 2.9 (SD 1.0) for the outcome scale of the PEQ and mean score of 5.0 (SD 1.2) for the emotional scale. If we assume an SD of 1.0 for the outcome scale and that a mean difference of 0.5 or more points between the intervention and control groups is of both clinically and practical importance, then to achieve 90% power for demonstrating this mean difference as being statistically significant at the 1% (two sided) level, this study would require 121 women per group (242 in total). Assuming that 20% of patients would not return completed questionnaires, the study aimed to recruit 304 patients (152 per group).

#### Randomisation

Randomisation occured on receipt of signed consent forms. Allocation was through stratified block randomisation. A senior statistician within ScHARR generated a randomisation schedule for these strata, using STATA software. This was held remotely by another research nurse, who was not directly involved in recruiting patients to this study. By referring to the list, she allocated patients to either the intervention group (ePAQ + telephone consultation) or the control group (standard care) and placed these randomised group numbers in a sealed envelopes. The randomised allocation enveloped were then passed on to the research nurse who opened them sequentially for each patient who consented to participate in the study. It was not possible to blind the clinicians or patients to the intervention. Those analysing the main study and cost-effectiveness outcomes were blinded until the point of data analysis.

#### Statistics and data analysis

Statistical analysis was conducted on an ‘intention-to-treat’ basis with the principal analysis directed to difference between post-consultation scores on the PEQ. A p-value ≤0.05 was regarded as statistically significant. As the study was a two-parallel group RCT, the study was reported according to the CONSORT guideline [28].

For the primary analysis, post-consultation PEQ scores were compared between the two arms (intervention and standard care), with analyses adjusted and unadjusted for covariates. The unadjusted analysis used a 2-independent samples t-test to compare mean post consultation PEQ scores between the two groups. A ninety-five percent confidence interval (CI) for the mean difference in post consultation scores between the two treatment groups was reported. The adjusted analysis used an analysis of covariance (ANCOVA) model with post consultation PEQ scores as the outcome and, age as a covariate. A 95% CI for the treatment group regression coefficient was reported.

Analysis of the post-consultation satisfaction scores followed the same format as the primary outcome. Due to the skewed distribution of the number of referrals the distributions were compared between the two arms (intervention and usual care) using Mann-Whitney U tests. The difference in proportion of patient referred and a ninety-five percent CI for the difference in proportion between the two treatment groups was reported. Multiple imputation using a regression model with age, BMI and parity was carried out on the primary outcome but found that it had no influence on the conclusions of the hypothesis tests.

## Results

**Fig 1:**
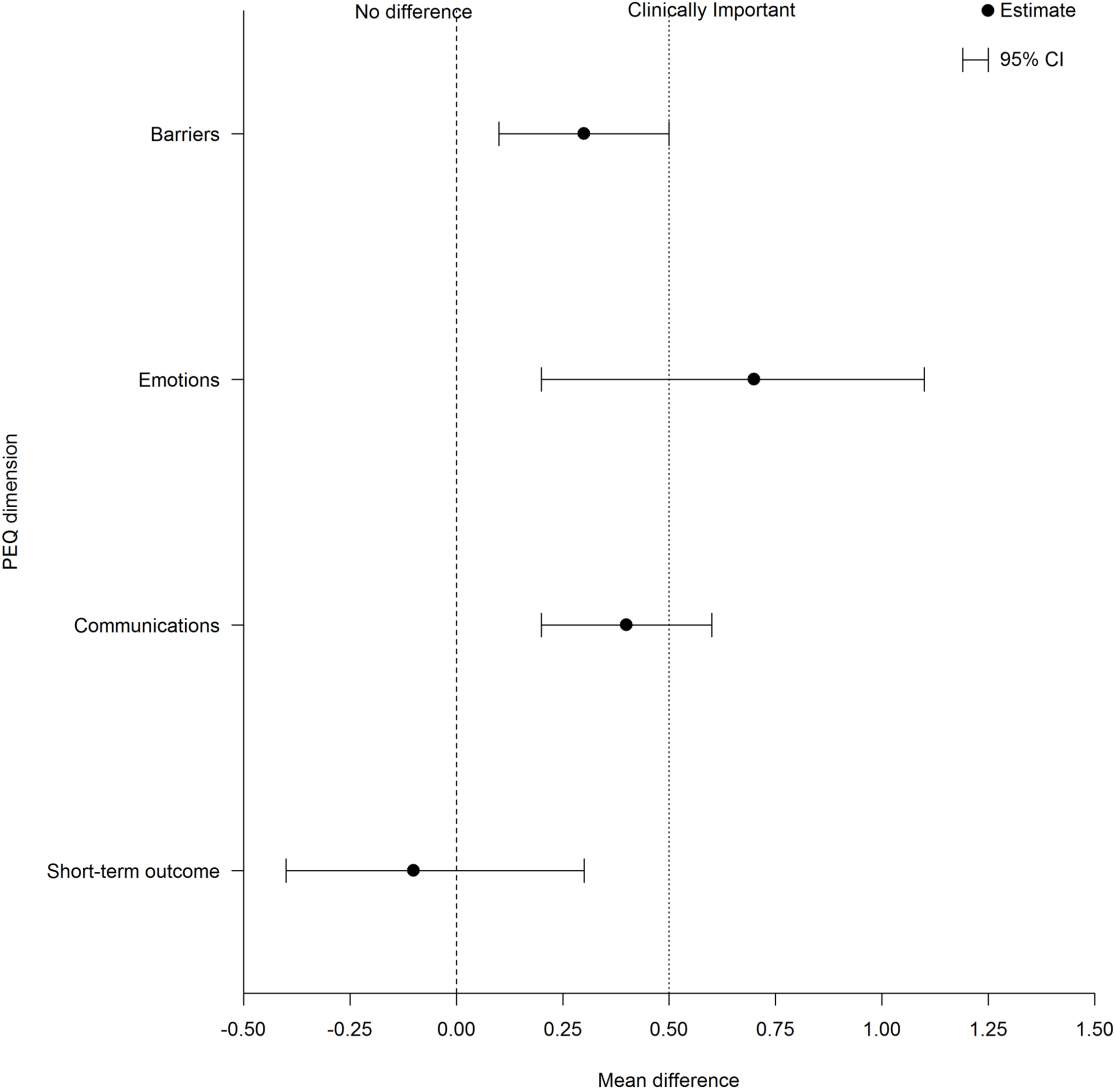
Participant progression through the trial—CONSORT flow chart.

A total of 515 referral letters were received but after screening by the clinical team for eligibility 434 women were telephoned about the study. Following the conversation with the research nurse, 293 women were sent the study information. The main reasons for declining participation included insufficient time to take part (e.g. patients had already been admitted to hospital), decided a different treatment pathway e.g. (private consultation), inappropriate (for reasons such as pregnancy, language barriers, learning difficulties, family circumstances, or other health problems such as cancer and deafness, and unsure if keeping the appointment). One hundred and ninety-five women returned the consent form and were randomised into the study. Ninety-eight women received the intervention and 97 received standard care (Fig 1).

However, six women in the intervention arm withdrew consent. Table 1 shows the baseline characteristics of the women who were randomised to the two groups. The mean age was 50.6 years in the control group (SD: 10.5; range 24.7–71.5 years) and 51.5 years in the intervention group (SD: 11.3; range 28.8–75.5 years). A comparison of baseline characteristics between those included in the primary analysis and those excluded from the primary analysis showed no real differences between the groups (S1 Table Baseline characteristics of those patients analysed vs. those not analysed by treatment group).

**Table 1:** Baseline characteristics of all randomised patients by treatment group.

		Control (n = 97)				Intervention (n = 98)			
		n	Mean	SD	Range	n	Mean	SD	Range
Age (years)		95	50.6	10.5	24.7–71.5	91	51.5	11.3	28.8–75.5
Height (m)		93	1.63	0.07	1.47–1.80	91	1.63	0.07	1.50–1.78
Weight (kg)		93	70.4	13.7	45.5–122.0	87	71.9	13.3	48.0–113.0
BMI		92	26.6	5.3	19.4–49.1	87	27.2	4.9	19.7 0 45.8
Parity		97	2		0–5	91	2		0–6
Nationality:	*American*	0	(0%)			1	(1%)		
	*British*	95	(98%)			88	(97%)		
	*European*	1	(1%)			1	(1%)		
	*Irish*	1	(1%)			1	(1%)		
Ethnic Origin:	*White British*	95	(98%)			86	(95%)		
	*White Irish*	1	(1%)			1	(1%)		
	*White Other*	0	(0%)			3	(3%)		
	*Mixed Race*	1	(1%)			1	(1%)		
Education Level:	*None*	17	(17%)			14	(16%)		
	*Junior*	26	(27%)			30	(33%)		
	*Senior*	27	(28%)			21	(23%)		
	*University*	27	(28%)			25	(28%)		
Marital Status:	*Married*	71	(73%)			64	(70%)		
	*Cohabiting*	8	(8%)			8	(9%)		
	*Single*	2	(2%)			4	(4%)		
	*Widowed*	5	(5%)			3	(3%)		
	*Divorced*	8	(8%)			10	(11%)		
	*Separated*	3	(3%)			1	(1%)		
	*Civil Partnership*	0	(0%)			1	(1%)		

There was no significant difference in the symptom profiles (as measured by ePAQ) in the two groups. The most prevalent conditions were stress incontinence and overactive bladder, and approximately 1/3 reporting sexual dysfunction. Women who were felt to need examination at their first assessment were excluded from the study, which is reflected in the low incidence of symptomatic and bothersome prolapse, reported by the women (Table 2).

**Table 2:** ePAQ domains scores for the study sample.

ePAQ-PF Domains	N	Mean Domain Scores—Virtual Clinic*	N	Mean Domain Scores—Standard Care*
**Urinary**				
Pain	88	11.8	89	11.5
Voiding	89	8.9	91	11.7
OAB	87	34.5	91	31.3
Stress	88	43.1	92	45.7
QoL	87	58.3		
**Bowel**				
IBS	88	26.5	92	25.4
Constipation	86	22.0	91	18.4
Evac	84	17.8	92	15.9
Continence	86	13.1	91	13.1
QoL	83	16.3		
**Vaginal**				
Pain & sensation	86	20.7	91	29.6
Capacity	82	4.7	86	5.3
Prolapse	87	13.4	89	18.4
QoL	83	15.2	89	15.4
**Sex**				
Urinary & Sex	75	26.3	81	29.0
Bowel & Sex	69	9.6	76	7.2
Vagina & Sex	74	23.7	79	27.0
Dyspareunia	74	21.7	79	21.2
Overall sex life	79	35.2	79	36.4

*NB: 0 = Perfect health, whereas 100 = worst health

The intervention group had a statistically significant difference in subsequent referrals to a general practitioner. The difference in proportions (and 95% CI) between treatment groups was 24.5% (6.8 to 42.1), p = 0.008 and the Mann-Whitney U test showed an overall difference in distribution (p = 0.015). The differences in subsequent referrals to practice nurse and outpatient between treatment groups were not statistically significant (Table 3).

**Table 3:** Number of referrals over 6 months by treatment group.

	Treatment Group												
	Control					Intervention							
	N	Proportion^1^	Total Referrals^2^	Median	Range	N	Proportion^1^	Total Referrals^2^	Median	Range	Difference in Proportion^3^ (95% CI)	P-Value^4^	P-Value^5^
General Practitioner	55	23.6%	33	0	0–10	52	48.1%	43	0	0–5	24.5% (6.8, 42.1)	0.008	0.015
Practice Nurse	55	10.9%	10	0	0–3	50	10.0%	7	0	0–2	-0.9% (-12.6, 10.8)	0.879	0.856
Outpatient*	55	63.6%	80	1	0–5	52	65.4%	76	1	0–9	1.8% (-16.4, 19.9)	0.850	0.818

^1^ Proportion of patients having at least one referral.

^2^Total number of referrals.

^3^A positive difference in proportion indicates the intervention has a higher proportion of referrals.

^4^P-Value from a Chi-Square test for difference in proportions.

^5^P-Value from a Mann-Whitney U Test comparing the distribution of referrals.

*Non-surgical hospital outpatient.

### Results: Patient experience

The primary outcome was the short term outcome score of the PEQ post consultation. This includes 4 questions relating to knowledge and understanding of a health problem comprising: 1. Do you know what to do to reduce your health problems? (or how to prevent further health problems?), 2. Do you know what to expect from now on?, 3. Will you be able to handle your health problems differently? and, 4. Will it lead to fewer health problems? (or help you to prevent such problems? This was available for 137 women (i.e. 70% of the original cohort) that could be analysed. There was no reliable evidence of a statistically significant difference in the short-term dimension between the control and intervention groups (Table 4).

**Table 4:** Unadjusted and adjusted differences in mean patient experience questionnaire scores post consultation by treatment groups.

	Control			Intervention			Unadjusted^2^				Adjusted^3^			
	N	Mean	SD	N	Mean	SD	Mean Diff	95% CI		P-Value	Mean Diff	95% CI		P-Value
PEQ dimension^1^								Lower	Upper			Lower	Upper	
Short-term outcome**	69	2.9	1.1	68	2.9	1.2	-0.04	-0.4	0.3	0.843	-0.03	-0.4	0.4	0.887
Communications	68	3.8	0.8	68	4.2	0.6	0.4	0.2	0.6	0.001	0.4	0.2	0.7	0.001
Emotions	66	4.6	1.3	64	5.4	1.2	0.7	0.2	1.1	0.001	0.7	0.3	1.1	0.002
Barriers	68	4.0	0.6	67	4.4	0.6	0.3	0.1	0.5	0.002	0.3	0.1	0.5	0.003

^1^The PEQ dimensions are scored on a 1–5 scale with the exception of emotions which is 1–7. A high score represents a good communication experience, positive emotions, positive consultation outcome and a lack of communication barriers.

^2^P-value from independent samples t-test.

^3^Adjusted mean difference calculated from a linear regression model with PEQ dimension score as the outcome and age, parity and treatment group as covariates. A positive mean difference indicates that the intervention group has the better score.

**This domain asks 4 questions:
Do you know what to do to reduce your health problems? (or how to prevent further health problems?) Do you know what to expect from now on? Will you be able to handle your health problems differently? Will it lead to fewer health problems? (or help you to prevent such problems?).

The unadjusted mean difference (and 95% CI) between treatment groups for the short term outcome was -0.1 (-0.4 to 0.3), p = 0.84. There was a statistically significant difference between the control and intervention groups for the other three dimensions of the PEQ (communications, emotions and barriers). The mean difference (and 95% CI) between treatment groups was 0.4 (0.2 to 0.6), p = 0.001 for the communications dimension, 0.7 (0.2 to 1.1), p = 0.001 for the emotions dimension and, 0.3 (0.1 to 0.5), p = 0.002 for the barriers dimension. These results remained after adjusting for the covariates of age and parity.

The age and parity adjusted mean difference (and 95% CI) between groups for the short term outcome was not significant -0.03 (-0.4 to 0.4), p = 0.887. There was a statistically significant difference between the control and intervention groups for the other three dimensions of the PEQ (communications, emotions and barriers). The mean difference (and 95% CI) between treatment groups is 0.4 (0.2 to 0.7), p = 0.001 for the communications dimension, 0.7 (0.3 to 1.1), p = 0.002 for the emotions dimension and, 0.3 (0.1 to 0.5), p = 0.003 for the barriers dimension (S1 Fig Mean difference between groups and 95% CI for post consultation PEQ scores).

### Results: Cost-utility analysis

The primary cost-effectiveness analysis was completed on patients with complete cost and outcome data (Control group N = 30; Intervention Group N = 27). Unit costs (S2 Table Unit Costs) were applied to resource use estimates (S3 Table Resource Use Within 6 Months Follow Up) for each treatment arm. Estimates of mean direct and indirect costs by intervention group can be seen in Table 5.

**Table 5:** Mean costs per patient by treatment group (complete case analysis).

Resource	Cost per patient Group 1 (Intervention) (£) N = 27	Cost per patient Group 2 (control) (£) N = 30	Mean Difference (£)	95% Confidence Interval Lower	95% Confidence Interval Upper	P-value
Cost of consultations						
Consultation cost^1^	29.35	69.52	-40.17	-	-	-
Cost of software	2.40	2.40	0	-	-	-
Cost of computer	N/A	0.25	-.25	-	-	-
**Total consultation costs per patient**	**31.75**	**72.17**	**40.42**	-	-	-
Direct costs during 6 month follow-up						
GP Visits	41.22 (49.49)	35.33 (65.78)	5.89	-25.29	37.06	.654
Practice nurse	0.94 (3.40)	2.13 (5.88)	-1.18	-3.77	1.41	.063
Outpatient visits	250.67 (316.09)	188.00 (246.547)	62.67	-87.02	212.36	.405
Cost of surgical procedures^2^	330.44 (707.375)	285.63 (784.36)	44.88	-353.35	442.97	.822
Other professionals						
Physiotherapist	5.04 (12.59)	4.99 (15.76)	.05	-7.58	7.68	.989
Specialist nurse (including stoma nurse, incontinence nurse, gynaecology)	4.52 (18.35)	2.03 (11.14)	2.49	-5.48	10.45	.534
Consultant (f2f)	7.94 (24.44)	14.29 (37.06)	-6.35	-23.22	10.51	.454
**Total direct costs**	**640.77 (844.40)**	**532.41 (867.09)**	**108.37**	**-346.93**	**563.67**	**.635**
Indirect costs during 6 month follow-up						
Personal expenditure in 6 month follow-up period (£)	24.07 (31.05)	16.17 (20.97)	7.9	-6.04	21.84	.261
Loss of productivity	443.26 (1573.15)	481.07 (1475.01)	-37.81	-847.04	771.42	.926
**Total indirect costs**	**467.33 (1569.42)**	**497.24 (1479.79)**	**-29.91**	**-839.47**	**779.66**	**.946**
**Total costs per patient**	**1,139.86 (2182.24)**	**1101.82 (2172.44)**	**38.04**	**-1119.34**	**1196.03**	**.948**

^1^Consultation cost includes consultant time and overheads.

^2^Healthcare Resource Group (HRG) codes included were validated by a clinician.

Consultation costs for the intervention group were less than half the costs of the control group (£31.75 versus £72.17), due primarily to the duration of the consultation and associated labour costs. The mean duration of the telephone consultation was 10.94 minutes, compared with a mean duration of 25.9 minutes for patients attending a face-to-face consultation.

Direct costs incurred during the 6 month follow-up period differed between treatment arms, with patients in the intervention group incurring non-significantly greater direct costs in comparison with the control group. This was driven primarily by the difference in costs associated with gynaecology outpatient attendances between arms, with those in the intervention group incurring costs of £62.67 greater than those in the control group.

Personal expenditure accrued at 6 month follow-up was higher in the intervention group However, lower costs associated with loss of productivity for the intervention group resulted in lower total (per-patient) indirect costs. The mean total cost per patient was estimated to be £1,139.86 for patients receiving the intervention and £1101.82 for the control group. This resulted in a non-significant mean differential cost of intervention versus control of £38.04.

Mean utility estimates by intervention are shown in Table 6. Within the intervention group, mean utility estimates reduced slightly from baseline to 6 months, whilst the equivalent estimates for Group 2 showed a slight increase. These estimates resulted in a non-significant QALY loss for patients in the intervention group relative to standard care of 0.0095 (p = 0.40).

**Table 6:** Mean utility per patient by intervention group (complete case analysis).

Item	Group 1 (Intervention) (N = 27)	Group 2 (Control) (N = 30)		95% CI of difference		
	Mean (SD)	Mean (SD)	Difference (SE)	Lower	upper	Significance
SF-6D baseline	.64 (0.090)	.62 (.081)	.026 (.024)	-.022	.07441	.287
SF-6D 6 months	.63 (.082)	.62 (.091)	.00698 (.023)	-.039	.05314	.763
Change in SF-6D	-.0152 (.073)	.0038(.094)	-.01899 (.02245)	-.06397	.02600	.401
QALYs gained	-.0076 (.037)	.0019 (.047)	-.0095 (.1122)	-.3199	.01300	.401

The cost-effectiveness acceptability curve (CEAC) for the complete case analysis (S2 Fig The Cost Effectiveness Acceptability Curve) indicates that under the commonly used funding threshold of £20k per QALY gained, the probability that the intervention is cost-effective is approximately 35%. After accounting for missing data through the use of multiple imputation the probability of cost-effectiveness increases to approximately 48% for the same threshold.

## Discussion

This randomised clinical trial investigated the effect of a ‘virtual clinic’ in urogynaecology on patient experience and cost. In terms of patient experience, the trial did not find any difference in the primary outcome (short term outcome dimension of the PEQ) between intervention and standard care, nor did the virtual clinic appear to affect general quality of life, satisfaction or women’s perceptions of using an online questionnaire system during their clinical episode. There was a statistically significant difference between the two study arms for the three other dimensions of the PEQ (communications, emotions and barriers). This finding suggests that for medical conditions of a sensitive and intimate nature, a positive element of implementing and using a virtual clinic includes the potential to better support patients to communicate health concerns, avoiding the embarrassment associated with describing and discussing intimate symptoms during a face to face consultation.

In clinical practice, patient assessment is central to diagnosis and management with a view to improving quality of life. It is well recognised that clinical interview data may be unreliable, being based on clinicians’ rather than patients’ views of their condition [29]. This is particulary relevant for sensitive areas such as urogynaecology, where computer or web-based formats have been found to result in greater disclosure [30]. The major advantage of administering PROMs electronically, compared with paper questionnaires, relate to the practicalities of clinical data capture; which can be superior in terms of efficiency and response rate and cost analysis has shown potential economic advantages [31–34]. It seems appropriate therefore, to seek ways of enhancing clinical assessment through well-designed and tested ePROMs in order to improve the quality of care and reliably measure outcome.

Whilst a comparison of a paper ePAQ with it’s electronic counterpart has not been carried out, our previous qualitative work has found the web-based version of value to patients, especially in terms of enhanced communication and preparedness for clinical consultation [14]. Our findings also suggest that a virtual clinic is a way of deploying web-based patient-reported outcome measures to support clinical consultations with potential benefits to patients in terms of experience and expense, although further evaluation in different clinical contexts is needed.

Overall, the standard care pathway was more cost effective, (52% chance that it was cost-effective based on the imputed analysis) which appeared to be the consequence of subsequent clinic attendance by a higher proportion of patients in this group. However, the trial found a significant difference between the duration of consultations (which were approximately 50% shorter in the intervention group) and associated consultation costs. The cost-effectiveness outcomes in the present study follow the same trend as shown in other studies. For example, Pinnock et al [35] found that telephone consultations for routine asthma reviews were less expensive than face-to-face consultations (£10.03 versus £12.74, mean difference £3.71; 95%CI = 1.92 to 3.50, P<0.001). Conversely, Beaver et al [3] performed an economic evaluation alongside a clinical trial, comparing hospital attendance with telephone follow-up after treatment for breast cancer and observed that patients receiving telephone follow-up had longer consultations, which was one reason for the resulting higher costs.

A strength of the current study was that it adopted a randomised trial design and thus was of high methodological quality and reduced risk of bias [36]. The study also recruited women attending routine clinical practice. However, due to the limited time of the grant award, only 195 of the target 304 participants were recruited and we were unable to extend recruitment, which is a limitation of the study. The lack of power also means that we cannot be certain there is no difference in the primary outcome, and the results should be treated with caution. Whilst recruitment ended in 2010 and thus the data is quite old now, we consider the results and our experiences of delivering a virtual clinic to still be of interest. Virtual clinics and the implementation of web-based technology is still very much a developing area in healthcare and there have been minimal changes to NHS tariffs and associated costs relevant to this study during the time since data collection ended.

The cost-effectiveness analysis suggests that the intervention is unlikely to be cost-effective at the £20,000 threshold. However, there are two problems with this simple interpretation. Firstly, as highlighted above, the inclusion of costs unrelated to the consultation introduces uncertainty and potential bias; the briefer consultations and lower unit costs, with associated patient satisfaction (communication) ratings, may be of value when considering different care pathways, particularly where further attendance, examination or investiagtions are unlikely to be required (e.g. surgical follow up). Secondly, whilst the SF-6D is considered the most appropriate generic utility measure for this type of intervention, it may not be reasonable to expect it to detect subtle effects of the intervention. Whilst patient experience and convenience could plausibly impact on wellbeing in the period immediately following an encounter with health services, it is unlikely that the SF6D woud detect this at six months. The cost-effectiveness acceptability curves are relatively flat, which demonstrate large amounts of uncertainty in both the costs and QALYs, and as such, are compatible with the view that the use of the SF6D is of little relevance in this context.

In light of these issues, it may be appropriate to take a more pragmatic approach that focuses on a broader range of benefits. This approach is termed a cost-consequences analysis [37] and is adopted in cases such as this when a simple incremental cost-effectiveness ratio (ICER) is not thought to capture all the relevant programme outcomes. If we adopt a more flexible stance that takes into account the wider effects of the intervention, we can see the potential value of this technology in reducing consultation costs and improving the communication experience of the patient.

The impact of treatment processes on well-being needs further consideration. Whilst several studies have identified so-called ‘process utility’, the methods by which this can be valued and incorporated into cost-effectiveness analysis is an under-researched area [38]. In situations where better patient experience is delivered at higher cost, decision makers need a way to guage whether or not the service change is worthwhile, given the opportunity cost of a compensatory service reduction elsewhere. Methods that can measure and capture process utility in a way that can be usefully included in cost-effectiveness analyses warrants further exploration and development.

## Conclusions

The virtual clinical had no impact on the short-term dimension of the PEQ and overall was not as cost-effective as standard care, which was attributed to subsequent clinic attendance by a higher proportion of patients in this group. However, for women referred to secondary care with urinary incontinence, the virtual clinic was associated with briefer consultation times, lower personal costs and enhanced communication. For medical conditions of a sensitive and intimate nature, a virtual clinic has potential to better support patients to communicate with health professionals about their condition. This approach does not appear to affect general quality of life, satisfaction or perceptions of using an online questionnaire system during their clinical episode.

To ascertain whether a virtual clinic can translate into genuine cost savings, future research is recommended in patient groups in whom subsequent additional clinic re-attendance (including additional GP attendance), is unlikely to be required, for example surgical follow-up.

## Supporting information

S1 Fig Mean difference between groups and 95% CI for post consultation PEQ scores (a positive mean difference indicates that the intervention group has a better score). (DOCX)

S2 Fig The Cost-effectiveness acceptability curve. (DOCX)

S1 Table Baseline characteristics of those patients analysed vs. those not analysed by treatment group. (DOCX)

S2 Table Unit costs. (DOCX)

S3 Table Resource use within 6 months follow-up. (DOCX)

S1 File Study protocol. (DOC)

S2 File CONSORT statement. (DOC)

## References

[1] Currell R, Urquhart C, Wainwright P, Lewis R. (2000) Telemedicine versus face to face patient care: effects on professional practice and health care outcomes. Cochrane Database Syst Rev. (2):CD002098 doi: 10.1002/14651858.CD002098 10796678 10.1002/14651858.CD002098

[2] Mark DA, Fitzmaurice GJ, Haughey KA, O’Donnell ME, Harty JC. (2011) Assessment of the quality of care and financial impact of a virtual renal clinic compared with the traditional outpatient service model. Int J Clin Pract. 10; 65 (10),1100–7. doi: 10.1111/j.1742-1241.2011.02750.x 21923849 10.1111/j.1742-1241.2011.02750.x

[3] Beaver K, Hollingworth W, McDonald R, Dunn G, Tysver-Robinson D, Thomson L et al (2009) Economic evaluation of a randomised clinical trial of hospital versus telephone follow-up after treatment for breast cancer. British Journal of surgery. 96(12), 1406–1415. doi: 10.1002/bjs.6753 19918858 10.1002/bjs.6753

[4] Hunter J, Claridge A, James S, Chan D, Stacey B, Stroud M et al (2012) Improving outpatient services: the Southampton IBD virtual clinic. Postgrad Med J. 8; 88 (1042), 487–91. doi: 10.1136/postgradmedj-2012-100123rep 22822228 10.1136/postgradmedj-2012-100123rep

[5] Levy S, Henderson L, McAlpine C. (2014) Growing up with confidence: using telehealth to support continence self-care deficits amongst young people with complex needs. Inform Prim Care. 21(3),113–7. doi: 10.14236/jhi.v21i3.58 25207614 10.14236/jhi.v21i3.58

[6] Tabak M, Brusse-Keizer M, van der Valk P, Hermens H, Vollenbroek-Hutten M. (2014) A telehealth program for self-management of COPD exacerbations and promotion of an active lifestyle: a pilot randomized controlled trial. Int J Chron Obstruct Pulmon Dis. 9(9),935–44. 25246781 10.2147/COPD.S60179 PMC4166347

[7] Dorsey ER, Venkataraman V, Grana MJ, Bull MT, George BP, Boyd CM et al (2013) Randomized controlled clinical trial of "virtual house calls" for Parkinson disease. JAMA Neurol. 5,70(5),565–70. doi: 10.1001/jamaneurol.2013.123 23479138 10.1001/jamaneurol.2013.123 PMC3791511

[8] Bhattacharyya R, Jayaram PR, Holliday R, Jenkins P, Anthony I, Rymaszewski L. The virtual fracture clinic: Reducing unnecessary review of clavicle fractures. Injury. 2017 3;48(3):720–723. doi: 10.1016/j.injury.2017.01.041 28168971 10.1016/j.injury.2017.01.041

[9] Connor A, Mortimer F, Higgins R. (2011) The follow-up of renal transplant recipients by telephone consultation: three years experience from a single UK renal unit. Clin Med. 6,11(3),242–6. 10.7861/clinmedicine.11-3-242 PMC4953316 21902076

[10] Radley SC, Jones GL. (2004) Measuring quality of life in urogynaecology. BJOG. 12,111 Suppl 1,33–6. Review. 15663154 10.1111/j.1471-0528.2004.00463.x

[11] Radley SC, Jones GL, Tanguy EA, Stevens VG, Nelson C, Mathers NJ. (2006) Computer interviewing in urogynaecology: concept, development and psychometric testing of an electronic pelvic floor assessment questionnaire in primary and secondary care. BJOG. 2,113(2),231–8. doi: 10.1111/j.1471-0528.2005.00820.x 16412003 10.1111/j.1471-0528.2005.00820.x

[12] Jones GL, Radley SC, Lumb J, Farkas A. (2009) Responsiveness of the electronic Personal Assessment Questionnaire-Pelvic Floor (ePAQ-PF). Int Urogynecol J Pelvic Floor Dysfunct. 5,20(5),557–64. doi: 10.1007/s00192-008-0790-9 19189036 10.1007/s00192-008-0790-9

[13] Jones GL, Radley SC, Lumb J, Jha S. (2008) Electronic pelvic floor symptoms assessment: tests of data quality of ePAQ-PF. Int Urogynecol J Pelvic Floor Dysfunct. 10,19(10),1337–47. doi: 10.1007/s00192-008-0655-2 18553041 10.1007/s00192-008-0655-2

[14] Dua A, Jones G, Wood H, Sidhu H. Understanding women’s experiences of electronic interviewing during the clinical episode in urogynaecology: a qualitative study. Int Urogynecol J. 2013 11;24(11):1969–75. doi: 10.1007/s00192-013-2132-9 23760093 10.1007/s00192-013-2132-9

[15] Schussler-Fiorenza SM, Gangnon RE, Chewning B, Wald A. (2015) Increasing Discussion Rates of Incontinence in Primary Care: A Randomized Controlled Trial. Journal of Women’s Health. Volume 24, Number 11 10.1089/jwh.2015.5230 PMC4649726 26555779

[16] Balas EA, Jaffrey F, Kuperman GJ, Boren SA, Brown GD, Pinciroli F et al (1997) Electronic communication with patients. Evaluation of distance medicine technology. JAMA. 7 9,278(2),152–9. 9214532

[17] Steine S, Finset A, & Laerum E. (2001). A new, brief questionnaire (PEQ) developed in primary care for measuring patients’ experience of health interaction, emotion and consultation outcome. Family Practice, 18(4), 410–417. 11477049 10.1093/fampra/18.4.410

[18] Larsen D.L, Attkisson C.C, Hargreaves W.A, and Nguyen T.D. (1979). Assessment of client/patient satisfaction: Development of a general scale, Evaluation and Program Planning, 2, 197–207. 10245370 10.1016/0149-7189(79)90094-6

[19] Moores KL, Jones GL, Radley SC. (2012) Development of an instrument to measure face validity, feasibility and utility of patient questionnaire use during healthcare: the QQ-10. Int J Qual Health Care. 24(5),517–24. doi: 10.1093/intqhc/mzs051 22879372 10.1093/intqhc/mzs051

[20] Ware JE, Kosinski M, and Keller SD. (1996) A 12-Item Short-Form Health Survey: Construction of scales and preliminary tests of reliability and validity. Medical Care, 34(3),220–233. 8628042 10.1097/00005650-199603000-00003

[21] Brazier JE & Roberts J. (2004) The Estimation of a Preference-Based Measure of Health from the SF-12. Medical Care. 42(9), 851–859. 15319610 10.1097/01.mlr.0000135827.18610.0d

[22] Brazier J, Ratcliffe J, Salomon JA and Tsuchiya A. (2007) Measuring and Valuing Health Benefits for Economic Evaluation. Oxford: Oxford University Press.

[23] Ramsey SD, McIntosh M, Sullivan SD. (2001) Design issues for conducting cost-effectiveness analyses alongside clinical trials. Annu. Rev. Publis Health. 22, 129–41. 10.1146/annurev.publhealth.22.1.129 11274515

[24] Husereau D, Drummond M, Petrou S, Carswell C, Moder D, Greenberg D et al, on behalf of the CHEERS Task Force. (2013) Consolidated Health Economic Evaluation Reporting Standards (CHEERS) statement. BMJ 346, f1049 doi: 10.1136/bmj.f1049 23529982 10.1136/bmj.f1049

[25] Curtis L. Personal Social Services Research Unit: Unit costs of Health and Social Care 2011. http://www.pssru.ac.uk/archive/pdf/uc/uc2011/uc2011.pdf Accessed 22nd February 2013.

[26] National Institute for Health and Clinical Excellence (2008). Guide to the methods of technology appraisal. http://www.pssru.ac.uk/archive/pdf/uc/uc2011/uc2011.pdf Accessed 22nd February 2013. 27905712

[27] National Schedule of Reference Costs 2011–02—NHS Trusts and PCTs Combined. Department of Health. https://www.gov.uk/government/publications/nhs-reference-costs-financial-year-2011-to-2012: Accessed October 2011.

[28] Altman DG, Moher D, Schulz KF. (2012) Improving the reporting of randomised trials: the CONSORT Statement and beyond. Stat Med. 10,31(25),2985–97.30 doi: 10.1002/sim.5402 22903776 10.1002/sim.5402

[29] Fitzpatrick R, Davey C, Buxton M, Jones DR. (1998) Patient-assessed outcome measures In: Black N., Brazier J., Fitzpatrick R., Reeves B., eds. *Health Services Research Methods*. BMJ Publications, London, 13–22.

[30] Schüssler-Fiorenza Rose SM, Gangnon RE, Chewning B, Wald A. Increasing Discussion Rates of Incontinence in Primary Care: A Randomized Controlled Trial. J Womens Health (Larchmt). 2015 11;24(11):940–9. 26555779 10.1089/jwh.2015.5230 PMC4649726

[31] Velikova G, Wright EP, Smith AB, et al (1999) Automated collection of quality of life data: a comparison of paper and computer touch-screen questionnaires. J Clin Oncol 117(3),998–1007. 10.1200/JCO.1999.17.3.998 10071295

[32] Kleinman L, Leidy NK, Crawley J, Bonomi A, Schoenfeld P. (2001) A comparative trial of paper-and-pencil versus computer administration of the Quality of Life in Reflux and Dyspepsia (QOLRAD) questionnaire. Med Care 39(2), 181–189. 11176555 10.1097/00005650-200102000-00008

[33] Buxton J, White M, Osoba D. (1998) Patients’ experiences using a computerized program with a touch-sensitive video monitor for the assessment of health-related quality of life. Qual Life Res 7(6), 513–519. 9737141 10.1023/a:1008826408328

[34] Lofland JH, Schaffer M, Goldfarb N. (2000) Evaluating health-related quality of life: cost comparison of computerized touch-screen technology and traditional paper systems. Pharmacotherapy 20(11), 1390–1395. 11079288 10.1592/phco.20.17.1390.34887

[35] Pinnock H, Adlem L, Gaskin S, Harris J, Snellgrove C. (2007) Accessibility, clinical effectiveness, and practice costs of providing a telephone option for routine asthma reviews: phase IV controlled implementation study. Br J Gen Pract 9 1,57(542),714–722. 17761059 PMC2151786

[36] Campbell MJ, Machin D, Walters SJ. (2007) Medical Statistics: A textbook for the health sciences. 4^th^ ed Wiley, Chichester, UK.

[37] Drummond MF, Sculpher MJ, Torrace GW, O’Brien BJ, Stoddart GL. (2005) Methods for the Economic Evaluation of Health Care Programmes. Oxford University Press, Oxford, UK.

[38] Brennan VK, Dixon S. (2013) Incorporating process utility into quality adjusted life years: a systematic review of empirical studies. Pharmacoeconomics. 8,31(8),677–91. doi: 10.1007/s40273-013-0066-1 23771494 10.1007/s40273-013-0066-1

