# Supplementary material for: Evaluating the impact of a ‘virtual clinic’ on patient experience, personal and provider costs of care in urinary incontinence: A randomised controlled trial
Source: PLoS One. 2018 Jan 18;13(1):e0189174. doi: 10.1371/journal.pone.0189174 (PMC5773012; doi:10.1371/journal.pone.0189174)
Supplement: S1 Fig — (DOCX) [file pone.0189174.s001.docx]

**S1 Fig: Mean difference between groups and 95% CI for post consultation PEQ scores (a positive mean difference indicates that the intervention group has a better score).**


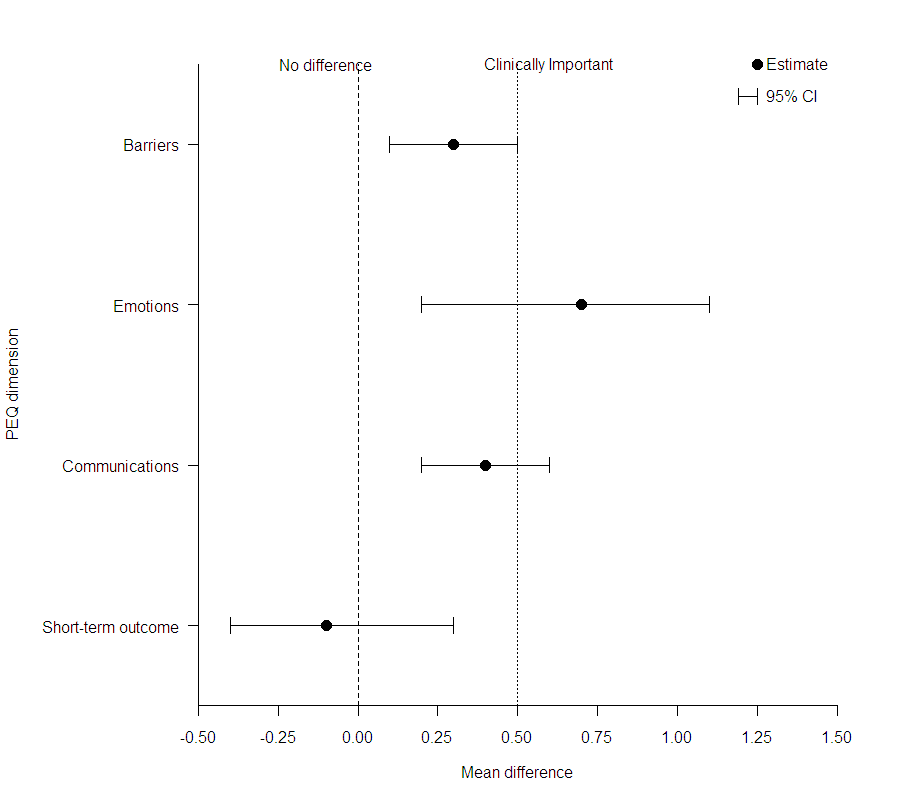


Supporting Information Table 1: Unit costs

| Resource Item | Unit | Unit Cost (£) | Source |
| --- | --- | --- | --- |
| General Practitioner Visit^1^ | Visit | 53 | Curtis (2011) |
| Specialist Nurse^2^ | Visit | 30.50 | Curtis (2011) |
| Practice Nurse^3^ | Visit | 12.75 | Curtis (2011) |
| Consultant (surgical)^4^ | Minute | 2.68 | Curtis (2011) |
| Physiotherapist^5^ | Visit | 13.60 | Curtis (2011) |
| Gynaecology OP | Outpatient visit | 141.00 | NHS Reference costs^6^ |
| Cost per day off work^7^ | Day | 88 | ONS 2011 |
| Computer cost in group 2 (control) | per patient | 0.25 | Personal communication from STH (SR) ^8^. |
| Cost of software | per patient | 2.40 | Personal communication from STH (SR) ^9^. |
| Consultant cost in group 1 (intervention) – including OH | per patient | 29.35 | Micro-costing study |
| Consultant cost group 2 (control) – including OH | per patient | 69.52 | Micro-costing study |
| Genital prolapse or incontinence | Per elective inpatient episode | 1,741 | HRG data MB02Z |
| Hysterectomy | Per elective inpatient episode | 3,346 | HRG data MA02Z |
| Bladder repair/bladder surgery | Per elective inpatient episode | 1,958 | HRG~MA04B |
| Botox to bladder | Per elective inpatient episode | 1,958 | HRG data MA04B |
| Lower genital tract disorders without CC | Per elective inpatient episode | 1,992 | HRG data MB01B |

1. Based on per clinic consultation lasting 17.2 minutes. Including direct care staff costs (table 10.8b Curtis 2011)
2. Based on Nurse Team Leader, £122 per hour of patient contact (including qualifications), duration of contact 15 minutes.
3. Based on Nurse (GP practice). £51 per hour of face-to-face contact. Including qualifications (table 10.6 Curtis 2011). Duration of contact 15 minutes.
4. Based on Consultant, surgical, hospital based. £161 per contract hour (Table 15.6 Curtis 2011)
5. Based on Hospital physiotherapist £35 per hour. Duration of contact for clinic appointment 23.3 minutes.
6. National reference costs 2010-2011, NHS trusts and PCTs combined consultation led: follow up attendance multiprofessional non-admitted face to face. Service code 502.
7. Based on 2011 Annual Survey of hours and Earnings. ONS Accessed on 01/011/2013 <http://www.ons.gov.uk/ons/dcp171778_256900.pdf>
8. Based on 2 touchscreen computers costing 1,000 per computer; average number of completions 1,000; computers replaced every 8 years. Annuity factor of 6.874.
9. Based on annual software cost of £2,400; average number of completions 1,000 per year.

Supporting Information Table 2: Resource use within 6 months follow-up

| Resource | Mean Resource Use Intervention (Group 1) | | Mean Resource Use Control (Group 2) | |  |
| --- | --- | --- | --- | --- | --- |
|  | N | Mean (SD) | N | Mean (SD) | Mean difference |
| GP Visits | 73 | .77 (1.112) | 78 | .65 (1.493) | -.113 |
| Practice nurse | 73 | .16 (.524) | 79 | .16 (.517) | -.002 |
| Outpatient visits | 74 | 1.59 (1.937) | 77 | 1.44 (1.509) | .153 |
| Mean number of surgical procedures | 74 | .220 (0.414) | 78 | .180 (.386) | .037 |
| Other professionals |  |  |  |  |  |
| Physiotherapist | 74 | .400 (1.030) | 77 | .450 (1.142) | -.056 |
| Stoma nurse | 74 | .010 (.116) | 79 | .000 (0.000) | .0140 |
| Incontinence nurse | 74 | .120 (.776) | 77 | .080 (.354) | .044 |
| Nurse specialist gynaecology | 76 | .030 (.161) | 78 | .000 (.000) | .026 |
| Consultant (f2f) | 75 | .170 (.601) | 77 | .260 (.616) | -.086 |
| Societal |  |  |  |  |  |
| Personal expenditure in 6 month follow-up period (£) | 74 | 27.944 (53.207) | 73 | 15.503 (27.666) | 12.441 |
| Time off work (days) | 70 | 3.09 0 (12.473) | 78 | 5.210 (20.763) | -2.119 |
| Time away from normal activities | 74 | 2.410 (8.039) | 79 | 1.290 (6.752) | 1.114 |

Supporting Information Figure 2: The Cost-Effectiveness Acceptability Curve.
