# Supplementary material for: Evaluating the impact of a ‘virtual clinic’ on patient experience, personal and provider costs of care in urinary incontinence: A randomised controlled trial
Source: PLoS One. 2018 Jan 18;13(1):e0189174. doi: 10.1371/journal.pone.0189174 (PMC5773012; doi:10.1371/journal.pone.0189174)
Supplement: S2 Fig — (DOCX) [file pone.0189174.s002.docx]

**S2 Fig: The Cost-Effectiveness Acceptability Curve.**
