# Supplementary material for: Evaluating the impact of a ‘virtual clinic’ on patient experience, personal and provider costs of care in urinary incontinence: A randomised controlled trial
Source: PLoS One. 2018 Jan 18;13(1):e0189174. doi: 10.1371/journal.pone.0189174 (PMC5773012; doi:10.1371/journal.pone.0189174)

**S1 Table: Baseline characteristics of those patients analysed vs. those not analysed by treatment group**


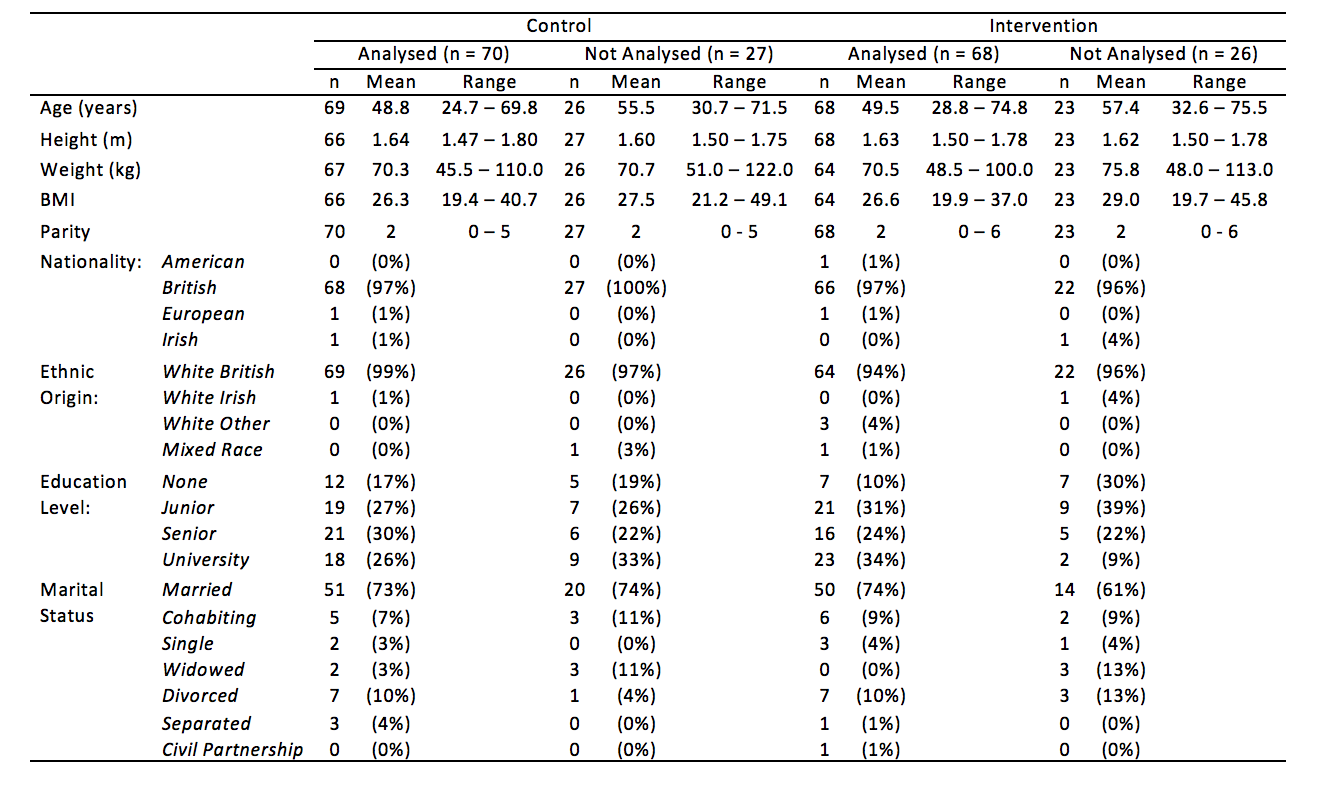

Supplement: S1 Table — (DOCX) [file pone.0189174.s003.docx]
