# Supplementary material for: Evaluating the impact of a ‘virtual clinic’ on patient experience, personal and provider costs of care in urinary incontinence: A randomised controlled trial
Source: PLoS One. 2018 Jan 18;13(1):e0189174. doi: 10.1371/journal.pone.0189174 (PMC5773012; doi:10.1371/journal.pone.0189174)
Supplement: S2 Table — (DOCX) [file pone.0189174.s004.docx]

S2 Table: Unit costs

| Resource Item | Unit | Unit Cost (£) | Source |
| --- | --- | --- | --- |
| General Practitioner Visit^1^ | Visit | 53 | Curtis (2011) |
| Specialist Nurse^2^ | Visit | 30.50 | Curtis (2011) |
| Practice Nurse^3^ | Visit | 12.75 | Curtis (2011) |
| Consultant (surgical)^4^ | Minute | 2.68 | Curtis (2011) |
| Physiotherapist^5^ | Visit | 13.60 | Curtis (2011) |
| Gynaecology OP | Outpatient visit | 141.00 | NHS Reference costs^6^ |
| Cost per day off work^7^ | Day | 88 | ONS 2011 |
| Computer cost in group 2 (control) | per patient | 0.25 | Personal communication from STH (SR) ^8^. |
| Cost of software | per patient | 2.40 | Personal communication from STH (SR) ^9^. |
| Consultant cost in group 1 (intervention) – including OH | per patient | 29.35 | Micro-costing study |
| Consultant cost group 2 (control) – including OH | per patient | 69.52 | Micro-costing study |
| Genital prolapse or incontinence | Per elective inpatient episode | 1,741 | HRG data MB02Z |
| Hysterectomy | Per elective inpatient episode | 3,346 | HRG data MA02Z |
| Bladder repair/bladder surgery | Per elective inpatient episode | 1,958 | HRG~MA04B |
| Botox to bladder | Per elective inpatient episode | 1,958 | HRG data MA04B |
| Lower genital tract disorders without CC | Per elective inpatient episode | 1,992 | HRG data MB01B |

1. Based on per clinic consultation lasting 17.2 minutes. Including direct care staff costs (table 10.8b Curtis 2011)
2. Based on Nurse Team Leader, £122 per hour of patient contact (including qualifications), duration of contact 15 minutes.
3. Based on Nurse (GP practice). £51 per hour of face-to-face contact. Including qualifications (table 10.6 Curtis 2011). Duration of contact 15 minutes.
4. Based on Consultant, surgical, hospital based. £161 per contract hour (Table 15.6 Curtis 2011)
5. Based on Hospital physiotherapist £35 per hour. Duration of contact for clinic appointment 23.3 minutes.
6. National reference costs 2010-2011, NHS trusts and PCTs combined consultation led: follow up attendance multiprofessional non-admitted face to face. Service code 502.
7. Based on 2011 Annual Survey of hours and Earnings. ONS Accessed on 01/011/2013 <http://www.ons.gov.uk/ons/dcp171778_256900.pdf>
8. Based on 2 touchscreen computers costing 1,000 per computer; average number of completions 1,000; computers replaced every 8 years. Annuity factor of 6.874.
9. Based on annual software cost of £2,400; average number of completions 1,000 per year.
