# Supplementary material for: Evaluating the impact of a ‘virtual clinic’ on patient experience, personal and provider costs of care in urinary incontinence: A randomised controlled trial
Source: PLoS One. 2018 Jan 18;13(1):e0189174. doi: 10.1371/journal.pone.0189174 (PMC5773012; doi:10.1371/journal.pone.0189174)
Supplement: S3 Table — (DOCX) [file pone.0189174.s005.docx]

S3 Table: Resource use within 6-months follow-up

| Resource | Mean Resource Use Intervention (Group 1) | | Mean Resource Use Control (Group 2) | |  |
| --- | --- | --- | --- | --- | --- |
|  | N | Mean (SD) | N | Mean (SD) | Mean difference |
| GP Visits | 73 | .77 (1.112) | 78 | .65 (1.493) | -.113 |
| Practice nurse | 73 | .16 (.524) | 79 | .16 (.517) | -.002 |
| Outpatient visits | 74 | 1.59 (1.937) | 77 | 1.44 (1.509) | .153 |
| Mean number of surgical procedures | 74 | .220 (0.414) | 78 | .180 (.386) | .037 |
| Other professionals |  |  |  |  |  |
| Physiotherapist | 74 | .400 (1.030) | 77 | .450 (1.142) | -.056 |
| Stoma nurse | 74 | .010 (.116) | 79 | .000 (0.000) | .0140 |
| Incontinence nurse | 74 | .120 (.776) | 77 | .080 (.354) | .044 |
| Nurse specialist gynaecology | 76 | .030 (.161) | 78 | .000 (.000) | .026 |
| Consultant (f2f) | 75 | .170 (.601) | 77 | .260 (.616) | -.086 |
| Societal |  |  |  |  |  |
| Personal expenditure in 6 month follow-up period (£) | 74 | 27.944 (53.207) | 73 | 15.503 (27.666) | 12.441 |
| Time off work (days) | 70 | 3.09 0 (12.473) | 78 | 5.210 (20.763) | -2.119 |
| Time away from normal activities | 74 | 2.410 (8.039) | 79 | 1.290 (6.752) | 1.114 |

m.abdelfattah@abdn.ac.uk
