# Supplementary material for: Evaluating the impact of a ‘virtual clinic’ on patient experience, personal and provider costs of care in urinary incontinence: A randomised controlled trial
Source: PLoS One. 2018 Jan 18;13(1):e0189174. doi: 10.1371/journal.pone.0189174 (PMC5773012; doi:10.1371/journal.pone.0189174)
Supplement: S1 File — (DOC) [file pone.0189174.s006.doc]

**Collaborating Organisations:**

|  |  |
| --- | --- |
| 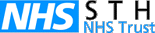 | **STHFT Department of Obstetrics & Gynaecology**  **&**  **University of Sheffield**  **Health Services Research Section, ScHARR** |

**Assessing the impact of using ePAQ on quality and cost of patient care in urogynaecology:**

**an RCT (STH14733)**

Study Protocol

**Proposed Duration Proposed Starting Date**

**24 months 01 December 2007**

**Members of the Research Team**

**Mr Stephen Radley**

Job Title: Consultant Gynaecologist

Organisation: Sheffield Teaching Hospitals

Department: Department of Obstetrics and Gynaecology

Full Address: Level 4, Jessop Wing Hospital

Phone Number: 0114 2268574

Fax Number: 0114 2268165

Email Address: [stephen.radley@sth.nhs.uk](mailto:stephen.radley@sth.nhs.uk)

**Dr Georgina Jones**

Job Title: Senior Lecturer

Organisation: University of Sheffield

Department: Health Services Research Section, ScHARR

Full Address: Regent Court, 30 Regent Street, Sheffield, S1 4DA

Phone Number: 0114 2220806/ 0114 2268515

Fax Number: 0114 2422136

Email Address: [g.l.jones@sheffield.ac.uk](mailto:g.l.jones@sheffield.ac.uk)

**Dr Swati Jha**

Post: Subspecialty Trainee in Urogynaecology

Organisation: Sheffield Teaching Hospitals NHS Trust

Department: Department of Obstetrics and Gynaecology

Full Address: Level 4, Jessop Wing Hospital

Phone Number: 0114 2268167

Fax Number: 0114 2268165

Email Address: [Swati.Jha@sth.nhs.uk](mailto:Swati.Jha@sth.nhs.uk)

**Dr Simon Dixon**

Job Title: Senior Lecturer

Organisation: University of Sheffield

Department: Health Services Research Section, ScHARR

Full Address: The University of Sheffield, Regent Court, 30 Regent Street, Sheffield S1 4DA

Phone Number: 0114 222 0724

Email Address: [s.dixon@sheffield.ac.uk](mailto:s.dixon@sheffield.ac.uk)

**Dr Stephen Walters**

Job Title: Senior Lecturer

Organisation: University of Sheffield

Department: Health Services Research Section, ScHARR

Full Address: The University of Sheffield, Regent Court, 30 Regent Street, Sheffield S1 4DA

Phone Number: 0114 222 0730

Email Address: s.j.walters@sheffield.ac.uk

**Professor William L. Ledger**

Job Title: Professor of Obstetrics and Gynaecology & Honorary Consultant in Obstetrics and Gynaecology.

Organisation: University of Sheffield

Department: Academic Unit of Reproductive & Developmental Medicine

Full Address: Room 5, Level 4 Jessop Wing Tree Root Walk Sheffield S10 2SF

Phone Number: 0114 226 8317

Email Address: [w.ledger@shef.ac.uk](mailto:w.ledger@shef.ac.uk)

**Title**

Assessing the impact of using ePAQ on quality and cost of patient care in urogynaecology: an RCT

**Background to the Research**

**What is ePAQ?**

ePAQ is a web-based interactive questionnaire (electronic pelvic floor assessment questionnaire) that provides a detailed evaluation of a woman’s pelvic floor symptoms and their impact on her quality of life. Touch-screen technology has been combined with computer programming (Dot Net & SQL Server) and existing paper-based instruments to produce an interactive user-friendly system that facilitates immediate data entry, analysis and storage. The instrument has evolved during several years' collaborative work with colleagues in a range of disciplines, including Gynaecology, Medical Physics, Colorectal Surgery, Urology and General Practice, as well as involving a women’s health user-group and regular surveys of patients' views.^1^ Research in primary and secondary care has established the psychometric properties of the instrument.^2^ The system has won a National award for innovation (HITEA) and was cited in Lord Warner’s Annual Report on NHS Innovation.^3^ EPAQ Systems Ltd is one of the first NHS spinout technology companies to be established in the UK (created jointly with Sheffield Teaching Hospitals NHS Trust in November 2006).

More recent development work has resulted in the creation of a website, where subjects can securely and anonymously access and use the ePAQ system via the Internet. This potentially allows subjects to use system in advance of clinic appointments (as opposed to the current practice of their completing it on arrival in clinic, immediately prior to their appointment). This study aims to evaluate the impact that using ePAQ in this way may have on the efficiency and quality of patient care. Initial survey data suggest that many women attending urogynaecology clinics do have access to the Internet and would be willing to use the system on-line & in advance of clinic appointments.^4^ The facility is also available in the Gynaecology Unit for women without Internet access to attend specifically for supervised use of the system. The in-depth assessment provided by the ePAQ may then be used to support an initial telephone consultation with a clinician, following which patients may be directed to the most appropriate clinic, as well as being provided with information and advice and have some forms of treatment initiated (such as behavioural therapy). The proposed randomised study aims to compare outcomes in women who use the ePAQ system in this way with women who undergo standard care in the Urogynaecology Unit.

The major advantage of electronic systems, when compared with paper questionnaires, relate to the practicalities of clinical data capture. Electronic questionnaires are comparable in terms of reliability and can be superior in terms of efficiency and response rate. In addition, electronic touch-screen questionnaires have been shown to be acceptable to patients, regardless of their age or educational background. Data quality is high, even in disadvantaged and technophobic subjects and cost analysis has shown potential economic advantages.^5,6,7,8,9^ Pelvic floor disorders in women, such as incontinence and prolapse, share common aetiologies and commonly coexist. Estimates vary, though it is estimated that approximately 20% of adult women experience regular urinary incontinence, 5% have some incontinence of faeces and 11% suffer with prolapse.^10,11,12^ However, despite better understanding of these conditions, bowel, bladder, vaginal and sexual dysfunction remain understandably taboo subjects and many women still regard them as inevitable consequences of childbirth and ageing. The personal cost to individuals is high, however simple and effective treatments are increasingly available. Many disorders respond well to behavioural, physical or medical therapy and such conservative treatments are generally recommended prior to consideration of invasive investigations or surgery.^13^ In clinical practice, clinical assessment is central to diagnosis and management and the restoration of function, with a view to improving quality of life, is the principal aim of treatment. It is well recognised that clinical interview data may be unreliable, being based on clinicians’ rather than patients’ views of their condition. It seems appropriate therefore, to seek ways of improving clinical assessment in order to enhance the quality of care and reliably measure outcome. Many women with pelvic floor disorders are managed in the community by GPs, nurses, physiotherapists or continence nurse advisors. In secondary and tertiary care, urologists, gynaecologists, colorectal surgeons and geriatricians are likely to be involved. However, at all levels, inconsistencies in clinical assessments represent an impediment to effective communication and the multidisciplinary approach advocated by the DoH, who in 2000 recommended the following:

• Full assessment leading to first line treatment in the primary care setting, with treatment & management plans agreed with individual patients.

• The provision of an integrated continence service, bringing together agreed protocols and procedures for primary, secondary and tertiary care.

• A comprehensive continence service, at home and in homes, bringing together all relevant health disciplines.

The further development of ePAQ has the potential to substantially augment this process by using on-line assessments to support patients' initial management and triage. Using ePAQ in advance of clinic appointments may provide patients and clinicians with prompt and valuable information and assist in directing patients to the most appropriate clinics in primary and secondary care. However, such a development warrants scrutiny in terms of patient experience and cost.

**3. Specific Research Question(s) / Hypotheses**

The ePAQ is now an established part of standard care & is in routine use in the Sheffield Urogynaecology Unit; All new patients are given the opportunity to complete the questionnaire on arrival in clinic and their printed results are then used to inform the subsequent clincial consultation. The aims of this research are to measure the impact that using ePAQ *in advance* of clinic appointments in combination with a telephone consultation has on patient care. Using an RCT methodology we will specifically evaluate its impact on the following:

**i) Patients’ experience of the clinical episode**

Outcome measures: Time to first assessment, Time to initiation of treatment, Patient Experience Questionnaire

**ii) Empowerment of patients in relation to their condition and healthcare that they receive**

Outcome measure: Modified Patient Enablement Index, QQ-10

**iii) Cost & efficiency**

Outcome measures: Number & type of clinic attendances & consultations, Costs of prescriptions & investigations

**4. Proposed research method.**

**a) Study design**

Randomised controlled trial. All women who consent to participate will be randomised to one of 2 groups:

1) ePAQ + telephone consultation

or

2) Usual care

**b) Description of the methodology**

This research will follow a quantitative methodology.

**c) Time frame**

24 months

Recruitment = 12 months. Follow-up = 6 months. Data analysis & preparation of results for publication = 6 months

**d) Sample**

All women referred to STH urogynaecology services, aged > 18 and able to read and understand English will be eligible to enter the study. Potential recruits to this study will be identified by review of referral letters received, from GPs or other clinicians, by the consultant to whom the patient has been referred (SC Radley or AG Farkas). It is envisaged that a minimum of 6 new patients per week will be recruited to this study.

**e) Sample size calculation**

The primary outcome for the purposes of sample size determination is the mean outcome scale score on the Patient Experience Questionnaire (PEQ) completed following the first consultation (Steine et al, 2001). 304 patients will be recruited (assuming a 20% dropout rate) to provide the 121 women needed in each group to detect significant differences between women who use ePAQ in advance of clinic appointments and women undergoing usual care (see Table 1 below).

**Table 1.**

Sample size for PEQ outcome scale at 1% and 5% significance levels and various standard deviations

|  | 1 | 2 | 3 | 4 | 5 | 6 |
| --- | --- | --- | --- | --- | --- | --- |
| *Test significance level* | 0.01 | 0.01 | 0.01 | 0.05 | 0.05 | 0.05 |
| *1 or 2 sided test?* | 2 | 2 | 2 | 2 | 2 | 2 |
| *Difference in means* | 0.5 | 0.5 | 0.5 | 0.5 | 0.5 | 0.5 |
| *Common standard deviation* | 1.2 | 1.1 | 1.0 | 1.2 | 1.1 | 1.0 |
| *Effect size* | 0.42 | 0.46 | 0.50 | 0.42 | 0.46 | 0.50 |
| *Power ( % )* | 90 | 90 | 90 | 80 | 80 | 80 |
| *n per group* | 174 | 146 | 121 | 92 | 77 | 64 |
| *Total N* | 348 | 292 | 242 | 184 | 154 | 128 |
| *Total N (with 20% dropout)* | 436 | 366 | 304 | 230 | 194 | 160 |

Steine (et al 2001) reported a mean score of 2.9 (SD 1.0) for the outcome scale of the PEQ and mean score of 5.0 (SD 1.2) for the emotion scale. If we assume an SD of 1.0 for the outcome scale and that a mean difference of 0.5 or more points between the intervention and control groups is of both clinical and practical importance, then to achieve a 90% power for demonstarting this mean difference as being statistically significant at the 1% (two sided) level will require 121 women per group (242 in total). Assumingthat 20% of patients do not return completed PEQs, then it will be necessary to recruit and randomise 304 patients (152 per group).

**Recruitment** (see flow chart: appx I)

Typically women are referred (by letter) by General Practitioners, other Hospital Specialists or Primary Care Continence Services. All referral letters will first be reviewed by the consultant (AGF or SCR) to whom the patient has been refferred. The consultant will then decide whether or not patients is suitable for study entry. Patients considered suitable by their consultant will be contacted by telephone by the Research Nurse to discuss the study (appx II). In order to prevent any significant delays in potential study patients being offered clinic appointments, patients who are not contactable by telephone within 48 hours of the reciept of the referral letter will be excluded from the study and be sent a routine outpatient clinic appointment. Patients who express no interest in participating in the study will also be sent a routine outpatient clinic appointment. Patients who express an interest in participating in the study will be sent an information leaflet about the study and a consent form (appendix III) with SAE. Women who do not respond to this within 1 week will be contacted by telephone to assess whether they wish to participate in the study. Those women who cannot be contacted at this point, or who decline study entry will be be sent a routine outpatient clinic appointment. On receipt of signed consent forms subjetcs will be entered into the study and randomised. At this point, patients randomised to standard care will be posted clinic appointments with AGF or SCR. Women randomised to ePAQ will be posted an information letter and epaq-online information leaflet inviting them to complete the questionnaire on-line and to telephone Gynae Appointments in order to arrange their telephone consultation (with AGF or SCR). Subjects in this group who are unable to complete epaq-online will use this telephone call with gynaecology appointments to arrange a separate visit to the hopsital to complete the questionnaire.

**f) Randomisation**

Randomisation will occur on receipt of signed consent forms. Allocation will be through stratified block randomisation. SW will generate a randomisation schedule, for these strata, using the STATA software. This will be held remotely by GLJ (who will not be directly involved in recruiting patients to the study). By referring to the list, she will allocate patients to either the intervention group (ePAQ + telephone consultation) or the control group (usual care). The randomised allocation will then be passed on to the research nurse who will then send the relevant information packs to each participant, relating to the arm of the study to which they have been randomised.

**Intervention Group: ePAQ clinic**

All women randomised to this arm of the study will be enetered onto the hospital PAS system under the ePAQ clinic. They will be invited by letter to complete epaq-online (and if necessary supervised ePAQ completion as a separate visit) and asked to contact Gynaecology Appointments in order to arrange their telephone consultation with AGF or SCR:

1. Letter inviting patients to complete epaq-online (including epaq-online information leaflet) & to telephone Gynaecology Appointments in order to book their telephone consultation.
2. Patient telephones Gynaecology Appointments when clerk will:
3. Ascertain whether patient has already succesfully completed epaq-online

If not: Agree & send a date, time & place to attend for supervised completion of ePAQ in hospital.

1. Agree & send (to all patients) a date, time & number for their telephone consultation
2. Scheduled telephone consultation with AGF or SCR

(supported by ePAQ report, referral letter and hospital casenotes)

1. Letter dictated to patient (including appropriate patient information leaflets & follow-up appointment)
2. Letter written to GP & referring clinician (including copy of above letter to patient)

The epaq-online information leaflet includes details of the web address for epaq-online (www.epaq-online.co.uk) with information explaining how to log-on and use the website. On completion of the questionnaire, the patient has the option of printing out a summary of her questionnaire data, which will include a summary of symptom scores in each area (urinary, bowel, vaginal and sexual). The patient is asked to forward her username and questionnaire ID to an STH email address (epaq@sth.hns.uk). Data are encrypted and anonymised (in line with good data protection practice) and can only be personalised to an individual patient by an approved clinician in receipt of the patient's username & questionnaire number, provided by the patient themselves. The clinician will use these results along with the patient's own casenotes and original referral letter to support the subsequent telephone consultation.

**Non-intervention (control) group: Usual care**

All women randomised to this arm of the study will be given an appointment to attend the urogynaecology clinic. 'Usual care' currently includes the option of completing the ePAQ on arrival in clinic, immediately prior to the clinical consultation. As is currently standard practice, the results of the ePAQ will be used to inform & support clinical assessment, however, as the ePAQ is used *immediately* prior to the clinical consultation, patients cannot be triaged or receive any additional information on the basis of their questionnaire results.

**Outcome Measures**

In order to achieve uniformity of approach (between ePAQ and control groups) outcome questionnaires (PEQ, PEI & QQ-10) will be posted to patients *immediately* after their first consultation. Patients will be asked to complete and return these by post.

**Primary Outcome Measure**

**Patient Experience Questionnaire**

The primary outcome for the purposes of sample size determination is the mean outcome scale score on the Patient Experience Questionnaire (PEQ) (Steine et al, 2001) (Appendix 6). The PEQ will be used to evaluate whether using ePAQ in combination with a telephone consultation results in any difference in the patient experience when compared with standard care. In order to evaluate the impact of using ePAQ in this way, we ask all patients (in both arms of the study) to complete the PEQ after their first clinical consultation.. The PEQ was developed and validated specifically to measure patients’ experience of interaction, emotion and consultation outcome. It contains 16 items in four dimensions, including 1) communication, 2) emotions 3) short-term outcome and 4) barriers. Three scales are scored from 1 to 5, and the emotion scale runs from 1 to 7. A high score represents a good communication experience, positive emotions, positive consultation outcome and a lack of communication barriers.

**Secondary Outcome Measures**

**1. Number and type of referrals**

The total number and individual type of referrals made will be used to evaluate the impact of ePAQ on the referral patterns and clinical visits made within the continence care pathway. At 6-month follow-up we will measure the number and type of referrals made on behalf of that patient; i.e. primary care (community nursing, community physiotherapy, GP, district nurse, practice nurse) and secondary care (gynaecology, urology, colorectal surgery, physiotherapy). These data will be collected in both groups using a standard proforma (appx VII).

**2. Number and type of prescriptions**

The number and type of prescriptions made will be collected to evaluate whether the use of ePAQ in advance of clinic appointments has any influence on the number of prescriptions made within the continence care pathway. These data will be collected at the 6-month follow-up on a proforma (appx VII) and supported by looking through the appropriate medical records. The prescribing of first line treatment will be categorised as (a) drug treatment (b) physiotherapy (c) behavioural therapy and (d) surgery.

**4. Modified Patient Enablement Index**

A modified version of the PEI will be administered (Howie et al, 1997; 1998) (Appendix 7). This validated instrument contains 6 items asking about the patient’s ability following a consultation to 1) cope with life 2) understand their illness, 3) cope with their illness, 4) keep themselves healthy, 5) feel confident about their health and 6) help themselves.

**5. Cost effectiveness**

The economic analysis will estimate the NHS costs of providing assessment and care up to six months in both arms of the study, and cost effectiveness by an incremental cost per quality-adjusted life year (QALY). These costs will include the costs of using ePAQ + telephone consultations, referrals, prescriptions (drugs / appliances / pads) and treatments. A broader societal costing will also be undertaken to consider costs to the patients and the economy in terms of private expenditure and time taken off work, respectively. These additional data will be in the standard proforma posted to both groups of patients at 6 months (appx VII).

Data on the use of the health services will be collected for each patient from records accessed from Trust and Primary Care practices. Data on private and societal costs will be gathered using patient-completed questionnaires at 6 months (appendix VII). Such patient-completed questionnaires are used in nearly all economic evaluations. Examples of questions include; "In the last 6 months, have you spent any money on anything because of bladder, bowel or vaginal problems?", "If yes, what did you spend it on?" "How much have you spent on each?", and "In the last 6 months, how many days have you needed to take off work because of bladder, bowel or vaginal problems and their treatment / care?" Costs will then be calculated using unit costs from routine sources (Curtis & Netten 2004, NHS Reference Costs and the British National Formulary). The costs associated with using ePAQ + telephone consulations compared with usual care will be estimated through a micro-costing study conducted during the first full 2-week period following recruitment of the 200^th^ patient to the study.

**h) Planned Analysis:**

All ePAQ data are held on a secure password protected database. All data will be anonymised and transferred to SPSS for Windows (version 14.0), which will be used to carry out all statistical analyses. As the trial is a parallel group RCT, data will be reported and presented according to the revised CONSORT statement (Moher et al, 2001). The statistical analyses will be performed on an intention-to-treat (ITT) basis. All statistical exploratory tests will be two-tailed with P < 0.05. Baseline demographic data will be assessed for comparability between the treatment groups. Other secondary outcomes are to be assessed at baseline (randomisation) and at 6 months post randomisation. In the event of differences between the ePAQ and usual care groups with respect to baseline demographic, physical, and health-related quality of life measurements, multiple regression or analysis of covariance (ANCOVA) will be used to adjust the treatment effect for these variables. The ordinary least squares adjusted regression coefficient estimate for the treatment group parameter along with its 95% confidence interval (CI) will then be reported.

**1. Analysis of the PEQ**

The primary efficacy response variable is the outcome scale of the PEQ. The PEQ is to be posted to the subject immediately after the consultation with the clinician. A two independent samples t-test will be used to compare mean PEQ outcome scale scores between groups (ePAQ and usual care) in this parameter. A 95% confidence interval (CI) for the mean difference in this parameter between the ePAQ and usual care groups will also be calculated. Secondary efficacy outcomes are the other four scales of the PEQ: communication; emotion; barriers and auxiliary staff. These will be analysed in a similar way to the outcome scale.

**2. Analysis of referral patterns**

At the 6 month follow-up we will measure (1) the number of referrals made for that patient and (2) the type of referral i.e. primary care (community nursing, community physiotherapy, GP, district nurse, practice nurse) and secondary care (gynaecology, urology, colorectal surgery, physiotherapy). The number of referrals over the 6-month follow-up will be described and tabulated. A two independent samples t-test will be used to compare mean number of referrals between the groups (ePAQ and usual care) in this parameter. A 95% confidence interval (CI) for the mean difference in this parameter between the ePAQ and usual care groups will also be calculated. The type of referral will be described and tabulated for each group. A chi-squared test will be used to compare referral patterns between the 2 groups (ePAQ and usual care).

**3. Analysis of the number of consultations**

The number and type of consultations over the 6-month follow-up period will be described and tabulated. An independent samples t-test will be used to compare mean number of consultations between the 2 groups (ePAQ in advance of clinic appointments vs usual care). A 95% confidence interval (CI) for the mean difference in this parameter will also be calculated.

**4. Analysis of the number and type of prescriptions**

Prescribing patterns (including number and type) over the 6-month follow-up period for each group will be described and tabulated. A two independent samples t-test will be used to compare mean numbers of prescriptions between the groups (ePAQ and usual care) for this parameter. A 95% confidence interval (CI) for the mean difference in this parameter between the ePAQ in advance of clinic appointments and usual care groups will also be calculated. A chi-squared test will be used to compare prescribing patterns between the groups (ePAQ and usual care).

**5. Analysis of cost-effectiveness**

The incremental cost-effectiveness ratio constructed from the cost and QALY data will be presented along with its associated cost-effectiveness acceptability curve. Separate analyses will be undertaken for NHS costs and the broader societal costs. One-way sensitivity analysis will be undertaken to assess the robustness of the results to changes in those variables where significant uncertainty remains, e.g. price of using ePAQ. Threshold analysis will also be undertaken to assess whether variation in any service characteristics exert significant influence on cost-effectiveness. These results can then be reformulated in a way that is clear to all service providers (Goodacre & Dixon 2005). Sub-group analysis will also be undertaken to assess the cost-effectiveness of using ePAQ in women of different age groups (e.g. under and over 50s) and with clinical conditions (e.g. incontinence and prolapse).

**6. Analysis of patient enablement**

The PEI is a six-item self-complete questionnaire, which generates a score with a 0 to 12 range, with a higher score indicating more patient satisfaction / enablement. The PEI is to be posted to patients in both arms of the study, immediately after their fist consultation. A 2 independent samples t-test will be used to compare mean PEI scores between the groups (ePAQ group vs and usual care) in this parameter. A 95% confidence interval (CI) for the mean difference in this parameter between the ePAQ and usual care groups will also be calculated.

**Reducing bias**

In this study, the elimination of bias is difficult, as neither patients nor clinicians (AFG & SCR) can be blinded to the group allocations following randomisation. Both AGF and SCR are involved in developing the urogynaecology service and regularly use the ePAQ system as part of routine clinical care. This will be fully acknowledged in any publications or presentations arising from this RCT. Between-group comparisons (AFG & SCR) will be made to look for systematic differences between these 2 consultant clinicians involved in this study. Both control and intervention groups will be given the opportunity to use the ePAQ: this instrument is currently established in routine care in the Urogynaecology Unit and it would be unethical and would potentially introduce bias to withdraw ePAQ use from the control group. Members of the research team (not involved in direct patient care) will carry out recruitment and randomisation of patients as well as the administration of outcome measures. Outcome will be measured in terms of both efficiency and quality, aiming to show whether any changes in efficiency (cost) are related to or at the expense of quality (patient experience) and vice versa. Control patients will use the ePAQ in clinic (as at present) immediately prior to their consultation. The intervention group will complete the questionnaire in advance of a telephone consultation held with their consultant (AGF or SCR). The use of standardised validated questionnaires as outcome measures (Patient Experience Questionnaire, Patient Enablement Index and QQ-10) will also provide generalisable benchmark data for the two groups of patients. Follow-up questionnaires (PEQ, PEI and QQ-10) will be posted to both groups immediately following completion of their first clinical consultation. The micro-costing study will take place over a 2-week period during the second half of the study. Clinicians conducting clinical interviews (in both arms of the study) by telephone and face-to-face will not be involved in issuing, collecting or collating outcome data.

**References**

1. Jha, S Radley, S,C., Jones, G.L. Patient experience of an electronic questionnaire in clinical practice (abstract). Poster: International Urogynaecology Association AGM, 2007
2. Radley, S,C., Jones, G.L., Tanguy, E.A., Stevens V.G., Nelson, C., Mathers N.J (2006) Computer interviewing in urogynaecology: concept, development and psychometric testing of an electronic pelvic floor assessment questionnaire (ePAQ) in primary and secondary care. British Journal of Obstetrics and Gynaecology 113(2):231-8.
3. Warner Report, NHS Information Authority, 6th January 2005: Modernisation & New Technology.
4. Radley, S.C. Jha, S. Jones, G.L. Bates, M. The Virtual Urogynaecology Clinic (abstract). Poster: International Urogynaecology Association AGM, 2007
5. Velikova G, Brown JM, Smith AB, Selby PJ. Computer-based quality of life questionnaires may contribute to doctor-patient interactions in oncology. *Br J Cancer* 2002; 86(1): 51-59.
6. Kleinman L, Leidy NK, Crawley J, Bonomi A, Schoenfeld P. A comparative trial of paper-and-pencil versus computer administration of the Quality of Life in Reflux and Dyspepsia (QOLRAD) questionnaire. Med Care 2001;39(2):181-189.
7. Thornberry J, Bhaskar B, Krulewitch CJ, Wesley B, Hubbard ML, Das A, Foudin L, Adamson M. Audio computerized self-report interview use in prenatal clinics: audio computer-assisted self interview with touch screen to detect alcohol consumption in pregnant women: application of a new technology to an old problem. Comput Inform Nurs 2002;20(2):46-52.
8. Adang RP, Vismans FJ, Ambergen AW, Talmon JL, Hasman A, Flendrig JA. Evaluation of computerised questionnaires designed for patients referred for gastrointestinal endoscopy. Int J Biomed Comput 1991;29(1): 31-44.
9. Arslanian C, Bond M. Computer assisted outcomes research in orthopedics: total joint replacement. J Med Sys 1999;23(3): 239-247.
10. Buxton J, White M, Osoba D. Patients' experiences using a computerized program with a touch-sensitive video monitor for the assessment of health-related quality of life. Qual Life Res 1998;7(6):513-519.
11. Jackson SL, Weber AM, Hull TL, Mitchinson AR, Walters MD. Fecal incontinence in women with urinary incontinence and pelvic organ prolapse. Obstet Gynecol 1997; 89(3):423-427.
12. Snooks SJ, Swash M, Henry M, Setchell M. Risk factors in childbirth causing damage to the pelvic floor innervation. Int J Colorectal Dis 2001;1(1): 20-24.
13. Muth S, Ramsay IN, Pringle S, Marahaj S, Tierney J. The incidence of unvoiced bowel problems in association with urinary incontinence in women. Abstract, ICS (UK) 10th Annual Meeting 2003: 35.
14. NICE Guideline on Incontinence. DoH 2006
15. DoH 2000 Advice on good practice for continence services
16. Curtis L, Netten A. Unit Costs of Health and Social Care 2004. Canterbury: University of Kent, 2004. Department of Health, London; NHS R&D Strategic Review: primary care. Report of Topic Working Group, June 1999.
17. Department of Health, London; Good practice in continence services. NHS
18. Executive, April 2000.Department of Health, London; The NHS Plan: a plan for investment, a plan for reform. NHS Command Paper, July 2000.
19. Department of Health, London; Delivering 21st century IT support for the NHS: national strategic programme. NHS, June 2002.Goodacre S, Dixon S. Is a chest pain observation unit likely to be cost-effective at my hospital? Extrapolation of data from a randomised controlled trial. Emergency Medical Journal 2005;22:418-422.
20. Howie JGR, Heaney DJ, Maxwell M. Measuring quality in general practice. London: Royal College of General Practitioners, 1997 (Occasional paper 75.)
21. Howie JGR, Heaney DJ, Maxwell M, Walker JJ. A comparison of a patient enablement instrument (PEI) against two established satisfaction scales as an outcome measure of primary care consultations. Fam Pract 1998;15: 165-171
22. Lofland JH, Schaffer M, Goldfarb N. Evaluating health-related quality of life: cost comparison of computerized touch-screen technology and traditional paper systems. Pharmacotherapy 2000;20(11):1390-1395.
23. Moher D, Schulz KF, Altman DG, for the CONSORT Group. The CONSORT statement: revised recommendations for improving the quality of reports of parallel group randomised trials. Lancet 2001; 357: 1191-94.
24. Steine S, Arnstein F, Laerum E. A new, brief questionnaire (PEQ) developed in primary health care for measuring patients’ experience of interaction, emotion and consultation outcome. Fam Pract 2001;18(4): 410-18.
25. Steine S, Arnstein F, Laerum E. A new, brief questionnaire (PEQ) developed in primary health care for measuring patients’ experience of interaction, emotion and consultation outcome. Fam Pract 2001;18(4): 410-18.
26. M, Brown J, Selby PJ. Automated collection of quality-of-life data: a comparison of paper and computer touch-screen questionnaires. J Clin Oncol 1999;17(3):998-1007.
